# Supplementary material for: Effects of Anti-Pseudomonal Agents, Individually and in Combination, With or Without Clarithromycin, on Growth and Biofilm Formation by Antibiotic-Susceptible and -Resistant Strains of Pseudomonas aeruginosa, and the Impact of Exposure to Cigarette Smoke Condensate
Source: Antibiotics (Basel). 2025 Mar 19;14(3):325. doi: 10.3390/antibiotics14030325 (PMC11939616; doi:10.3390/antibiotics14030325)
Supplement: Supplementary file 1 [file antibiotics-14-00325-s001.zip › antibiotics-3515517-supplementary.pdf]

SUPPLEMENTARY MATERIAL

Legend to the Figures.

Supplementary Figure S1. The effect of varying concentrations of cigarette smoke condensate (CSC) on the three strains of *Pseudomonas aeruginosa* (*Psa*) in (a) planktonic and (b) biofilm-forming cultures. The results are of three separate experiments performed in duplicate. Statistically significant values are shown by the asterisk (*) representing p values < 0.05. (a) The effect of varying concentrations of CSC on growth of bacterial strains in planktonic culture. For the wild-type (WT) strain, significantly increasing growth in planktonic cultures was found at concentrations of CSC of 3.12 and 6.25 mg/mL (p values of < 0.016 and 0.04, respectively). For the drug-sensitive (DS) strain, significantly increased growth in planktonic cultures was documented in concentrations of 100, 200 and 400 mg/L (p values 0.01, 0.03 and 0.002, respectively), while for multidrug-resistant (MDR) strains significantly increased growth was seen at concentrations of 12.5, 25, 50, 100, 200 and 400 mg/L (p values 0.03, 0.03, 0.01, 0.02, 0.002, and 0.004, respectively). (b) The effect of varying concentrations of CSC on growth of bacterial strains in biofilm-forming cultures. For the WT strain significantly increased growth occurred for concentrations of 25 mg/L (p value 0.04), for MDR strain this was 400 mg/L (p value 0.002), while there was no other CSC concentrations that resulted in significant effects for any of the strains. Abbreviations: CSC, cigarette smoke condensate; DS, drug-sensitive; MDR, multidrug-resistant; OD, optical density; *Psa*, *Pseudomonas aeruginosa*; WT, wild-type.

Supplementary Figure S2. The effect of varying concentrations of cigarette smoke condensate (CSC) on preformed biofilm of the wild-type (WT) strain. The results are of three separate experiments performed in duplicate. Statistically significant values are shown by the asterisk (*) representing p values < 0.05. While for concentrations between 0.4 and 12.5 mg/L there appeared to be a significant increase in biofilm (p values between 0.002 - 0.005), for higher concentrations between 100 - 200 mg/L there was a decrease in biofilm formation (p value of 0.04). Abbreviations: CSC, cigarette smoke condensate;

DS, drug-sensitive; MDR, multidrug-resistant; OD, optical density; *Psa*, *Pseudomonas aeruginosa*; WT, wild-type.

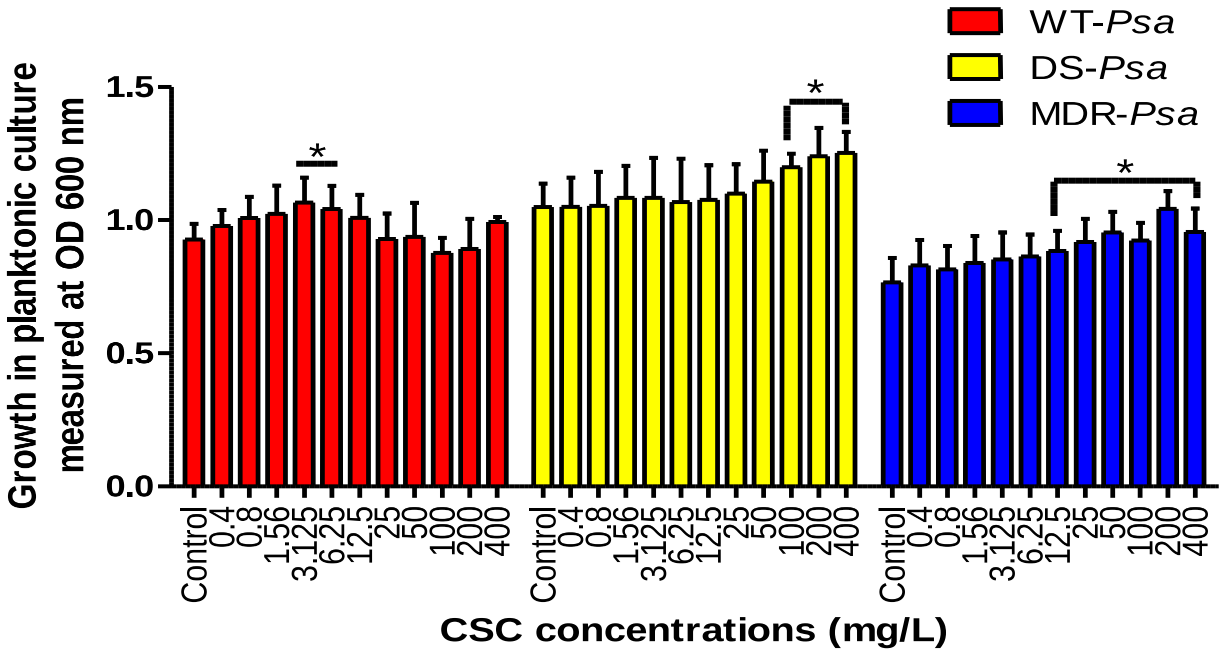

(a)

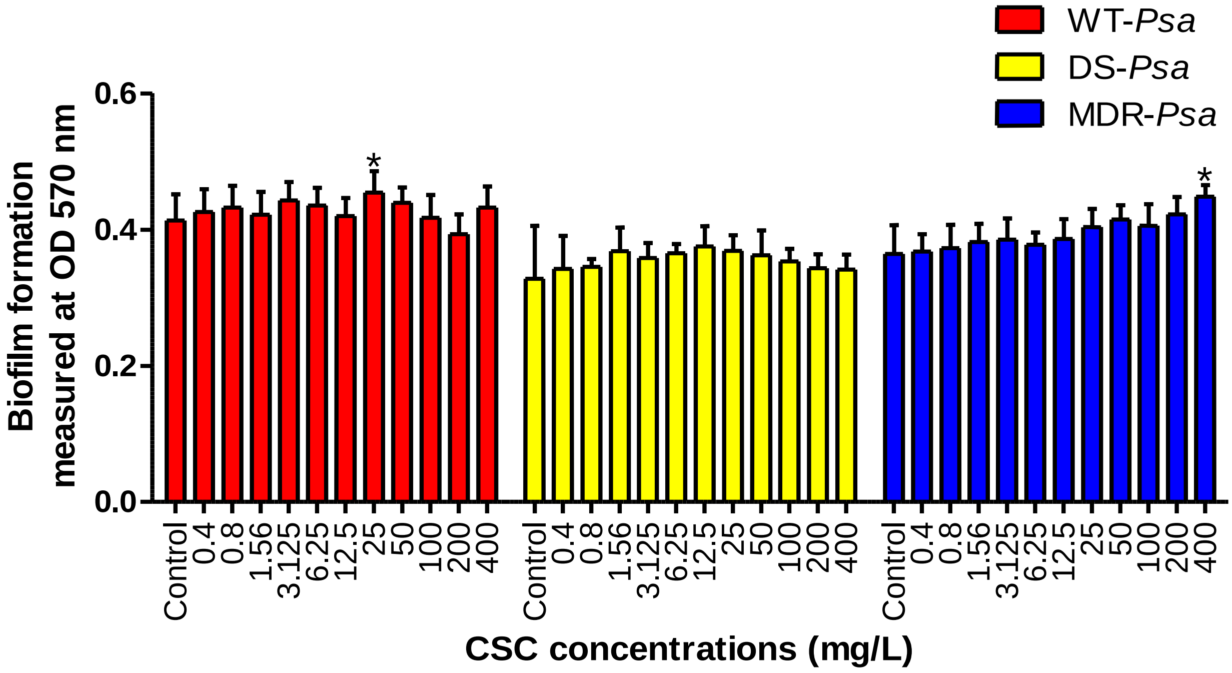

(b)

Supplementary Figure S1. The effect of varying concentrations of cigarette smoke condensate (CSC) on the three strains of *Pseudomonas aeruginosa* (*Psa*) in planktonic and biofilm-forming cultures.

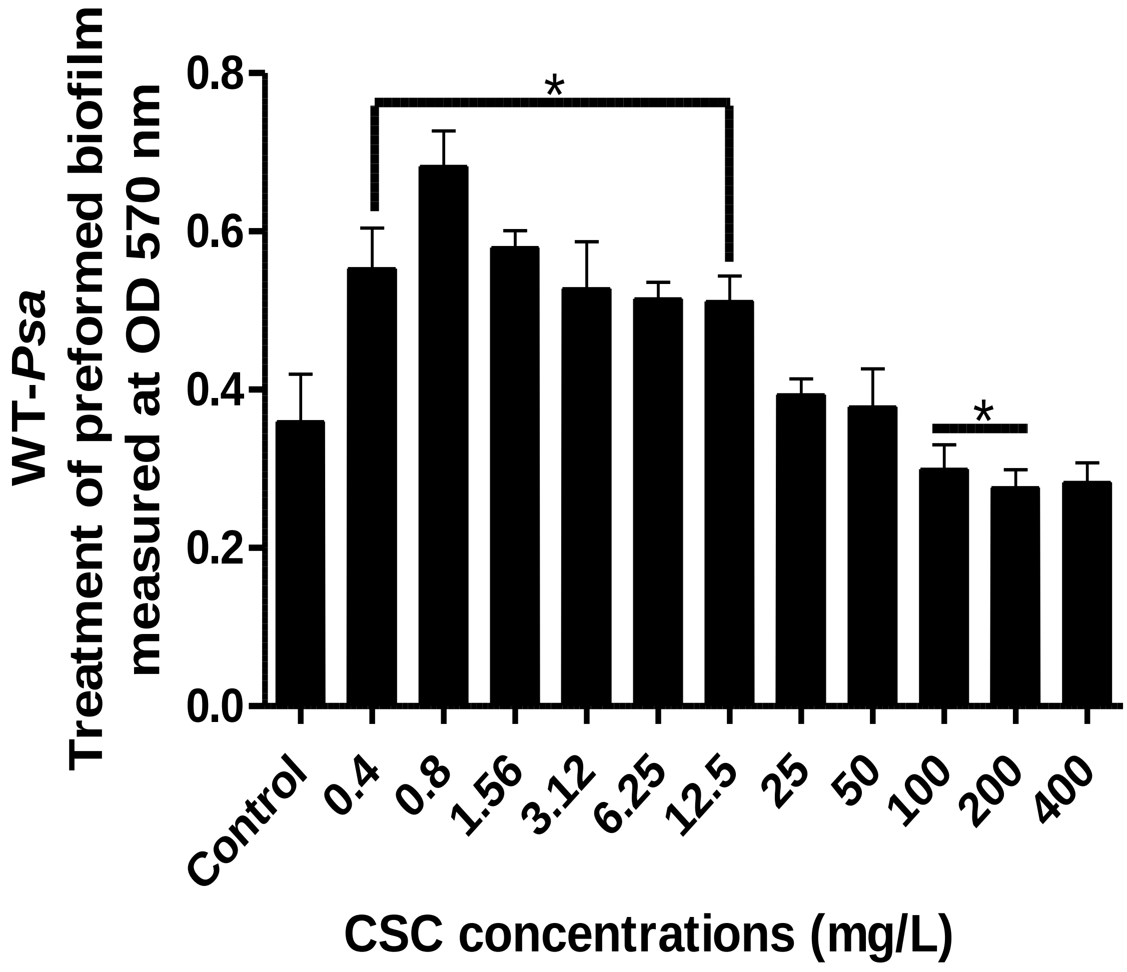

Figure S2. The effect of varying concentrations of cigarette smoke condensate (CSC) on preformed biofilm of the wild-type (WT) strain.
